# Cook County Medical Society, March 1st

**Published:** 1853-03

**Authors:** 


*Cook County Medical Society, March 1st.*

Dr. J. H. Bird called the attention of the society to the use of sugar of milk as a nutritious agent. He reported two cases of children fed with cow's milk, in which the structures composed of the protein compounds were rapidly developed, while the fatty tissues were atrophied to a remarkable degree. In both these cases he added to the ordinary food the sugar of milk in liberal quantities, and was gratified to find that the vice of nutrition was corrected. He suggested that the milk of the cow contains more casein and less sugar than the milk of the human female, and that by the use of this substance the natural nourishment of the infant is approximated at least. Dr. B. was led to prefer the sugar of milk to cane sugar from the fact that the former is readily converted into lactic acid, and serves as respiratory food; while, from recent experiments, it is probable that the latter acts as a stimulant to the kidneys, and is excreted through those organs.

Dr. Davis has observed that children who do not do well on the milk of the cow often become healthy and grow fat on the addition of common sugar—thinks the sugar of milk may be preferable; at all events it is worthy of trial.

Dr. Palmer has never used it, but likes the suggestion and thinks the subject important and worthy of further investigation.

Dr. Johnson thought the sugar of milk might be absorbed without being digested, and for that reason valuable as an article of food in disease of the alimentary canal or mesentery glands—thought it might be found a valuable substitute in some cases for cod-liver oil.

Dr. Herrick reported a case of disease of the lymphatic and mesenteric glands, in which it was almost impossible to introduce sufficient food to keep the patient alive; cod-liver oil had been used, with good albuminous diet and the various preparations of Iron, but without improvement; as a last resort the sugar of milk was used freely—the patient has been much better since. How much influence the treatment had in ameliorating the symptoms, it is impossible to say, but is strongly impressed with the idea that the use of the article has been beneficial.

Dr. Davis reported a case of unusual development of the fœtus in utero at the expense of the mother. Dr. Herrick reported cases of a similar nature, and also of unequal distribution of food during lactation—abortion in the one case and weaning in the other the only means of saving the life of the mother.

Dr. Davis also reported a case in which unusual symptoms followed the external application of infusion of aconite ℥ss. of the leaves to Oj. of water. The patient seemed to be laboring under extreme depression similar to that occasioned by fright.

Dr. Haven gave an account of a case of hydrophobia which had recently occurred in the city. The patient had been bitten about three weeks before taken—lived but three or four days. J.

